# Niche formation of *Rozellomycota* in wastewater treatment model reactors

**DOI:** 10.1038/s41598-025-23542-6

**Published:** 2025-10-15

**Authors:** Katrin Stüer-Patowsky, Eva Gega, Lijia Cao, Christian Wurzbacher

**Affiliations:** 1 Chair of Urban Water Systems Engineering, Technical University of Munich, Am Coulombwall 3, 85748 Garching, Germany; 2 Sanitary Engineering and Waste Management, University of the Bundeswehr Munich, Werner-Heisenberg-Weg 39, 85577 Neubiberg, Germany

**Keywords:** Aquatic fungi, Cryptomycota, DHS reactor, Freshwater ecology, Microbial communities, Environmental microbiology, Fungi, Civil engineering

## Abstract

A comprehensive understanding of microbial processes is a vital objective of wastewater treatment research efforts. The fungal phylum *Rozellomycota* is consistently present in relatively high abundances in wastewater treatment plants, being one of the dominant eukaryotic organisms. However, their impact is unknown. In this study, we aim to enlighten the abundances and roles of *Rozellomycota* as dominant eukaryotes in wastewater treatment model reactors. Replicated down-flow hanging sponge reactor systems, chemical water parameter surveillance, quantitative and qualitative DNA and cDNA analysis, and abundance correlations were used. *Rozellomycota* exhibited around 50 distinguishable 18S sequence variants as the second most abundant group, next to ciliates. They don’t show a significant gradient over the height of the reactors, being abundant in all samples from day 6 onwards, but a temporal succession within the reactor biofilms. The highest diversity of *Rozellomycota* was found between operation days 13 and 27. Within the experiment, we found single correlations between *Rozellomycota*, *Bacteria,* and *Ciliophora*, pointing to biotic linkages. Furthermore, we found a negative correlation with nitrogen. The high abundance of *Rozellomycota* in all samples indicates their distinct role in wastewater biofilms. Those insights into the complex communities can ultimately help optimize wastewater treatment processes.

**Supplementary Information:**

The online version contains supplementary material available at 10.1038/s41598-025-23542-6.

## Introduction

Fungi are known to be dominant in performing carbon degradation in the terrestrial environment, but their role in aquatic ecosystems is largely uninvestigated. Nevertheless, recent studies showed that fungi are abundant in various aquatic habitats. The *Cryptomycota*^1,2^, or *Rozellomycota*^3,4^, a rather newly discovered fungal phylum, is particularly interesting due to its abundance in engineered biological systems like wastewater treatment plants (WWTPs). They appeared to be the dominant eukaryotic organisms in anaerobic WWTP environments^5,6^ or in anaerobic or aerobic trickling filter-like systems^7^.

*Rozellomycota* are closely related to *Aphelidea*^8^ and *Microsporidia*^3^. Tedersoo et al.^9^ even proposed joining *Microsporidia* and *Rozellomycota* under one phylum. Whereas *Microsporidia* infect *Metazoa* and some protists, and *Aphelidea* target mainly algae^8,10^, *Rozellomycota* are known parasites of fungi such as *Chytridiomycota* and *Oomycota*^11^. The clade LKM15 is also proposed to fall within the phylum *Rozellomycota*^12^. As only specialists are able to live under anaerobic conditions (e.g. the anaerobic gut fungi^13^ and fermentative fungi like yeasts), it is unclear what *Rozellomycota* in wastewater systems do and how they cope with the very low environmental redox potential. Information on interactions, association with other organisms, and overall role in wastewater of *Rozellomycota* are, although highly abundant, is still missing.

In the study by Miyaoka et al.^7^, the application of a down-flow hanging sponge (DHS) system enabled the enrichment of fungi with a dominance of *Rozellomycota*. The aggregation of biomass and the formation of biofilms provided a positive selection pressure for the organisms of interest. The working principle of DHS reactors is similar to that of trickling filter systems^14^. In contrast to these systems, polyurethane foam sponges are used as filling media, which enables a three-dimensional space for sludge retention and growth of microorganisms in high biomass concentrations. The system is well adapted for sewage treatment and was developed to overcome the shortcomings of the activated sludge process^14^. It is mainly used as a posttreatment of up-flow anaerobic sludge blanket (UASB) reactors by providing efficient carbon degradation, ammonium oxidation, denitrification, and pathogen removal while retaining sludge in higher concentrations than conventional activated sludge systems^14,15^. The overall concept of this system enables the dissolving of air into water without external aeration, which reduces operating costs. Furthermore, polyurethane foam is a low-cost medium providing the needed characteristics like insolubility, resistance against biodegradation, high mechanical stability, and a high void ratio^16^. DHS reactors are easy to install and maintain on a low budget, showing stable performance^17^. As the DHS System showed an enriched *Rozellomycota* relative abundance^18^, it should be possible to closely investigate the occurrence and succession of *Rozellomycota* during the reactor start-up and operation phases.

This study aims to gain detailed insight into the abundance and structure of *Rozellomycota* communities in comparison with the rest of the microbial community. We are particularly interested in *Rozellomycota’s* role in biological wastewater treatment using DHS trickling filter reactors as model systems. *Rozellomycota* fungi have been exclusively described as parasites of other eukaryotes, mainly fungal phyla^11,19^. Therefore, their role in DHS reactors could be similar, altering the carbon flux by parasitism of, e.g., fungi. To monitor *Rozellomycota* in detail, we recently developed a quantitative PCR system to target the *Rozellomycota* molecular abundances^20^ in the DHS system, which we can now use in combination with community composition changes to inspect the changes of *Rozellomycota*, Eukaryotes, and Bacteria over time and the correlations with physicochemical parameters. We expected gradual growth phases of the biofilm following the temporal and spatial gradient, with fungi showing delayed growth. Further, an active selection of fungi to an extent where *Rozellomycota are* the dominant group of eukaryotic organisms, showing a high diversity, was expected. The prevalence of *Rozellomycota in* wastewater treatment systems may lead to a new understanding of this fungal lineage. Our results will help to better understand the eukaryotic component of solid-bed biological wastewater treatment approaches and will guide future research.

## Methods

### DHS reactor design

**Fig. 1:**
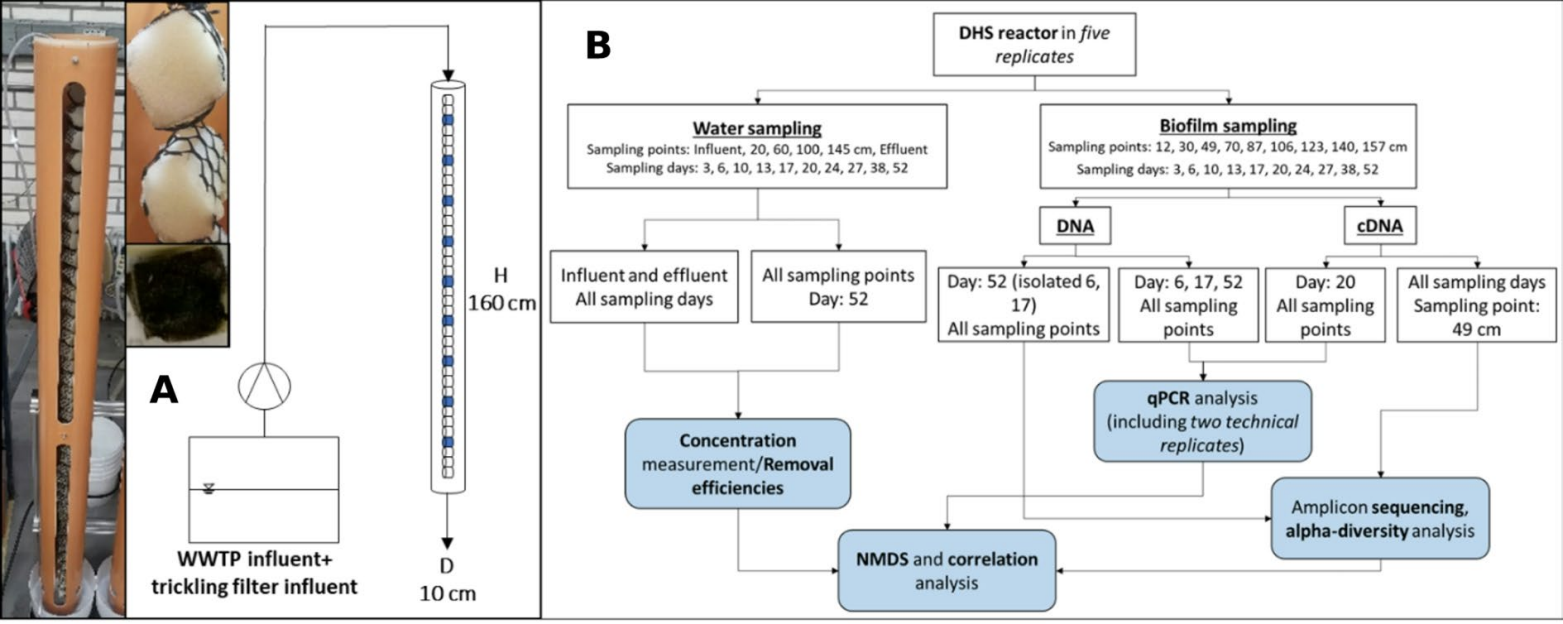
(**A**) Photograph and sketch of the experimental set-up of one of the five parallel DHS-reactors, including a picture of clean and overgrown polyurethane foam sponge filling. (**B**) Overview of sampling and analysis scheme.

The experimental set-up consisted of five parallel DHS reactors (Fig. 1A), each filled with strings of 38 3*3 cm polyurethane foam sponges assembled using stiff polyethylene netting and cable ties. To minimize the exposure of the carriers to light and movement in the experimental hall, 160 cm long water pipes with a diameter of 10 cm were added around them. For subsequent water quality analysis, effluent was collected at the base using individual basins. Nine sponges over the height of the reactors (12, 30, 49, 70, 87, 106, 123, 140, and 157 cm from the top; height measured in wet state with stretched filling strings) were chosen as sampling points for DNA (spatial sampling) and RNA extraction (temporal sampling). As a feed, a 2:1 mixture of the influent of the communal WWTP in Garching after the sand trap and the influent of the trickling filter reactors of the same WWTP was used. Before the reactor started, the filling was inoculated with trickling-filter effluent of the communal WWTP in Garching. The reactors operated under continuous flow conditions (HRT = 7.2 h, Q = 2.6 ml/min) for 52 days.

### Water sampling and chemical parameter measurement

Two times a week for five weeks (on days 3, 6, 10, 13, 17, 20, 24, 27, 38, and 52 of operation), samples of the influent and the effluent of each reactor were taken (Fig. 1B). Temperature and pH were measured immediately on-site, and samples were subsequently stored at 4 °C until analysis. Chemical parameters were analysed using standardized test kits: COD (LCK314, Hach), ammonium (LKC303/LKC304, Hach), total nitrogen (LKC138, Hach), and BOD (DIN EN 1899-1, DEV H51). On the last sampling day, additional water samples were taken at 20, 60, 100, and 145 cm from the top of the reactor. The determination of nitrate (LKC 339/LKC340, Hach) was added to the analysis, while BOD was omitted for those samples. To evaluate the change of the water parameters within the reactors and classify the wastewater treatment effect, removal efficiencies were calculated, following the equation:

$$E(\%) = ({c}_{in} - {c}_{out})/{c}_{in} x 100$$

### Sponge sampling and RNA and DNA extraction

Sponge samples were collected on the same days as the water samples. A defined sponge mass of 43.88 mg was taken by prepared punch-outs to extract DNA and RNA. The received sponge material was cut into small pieces to prepare for the bead-beating step of the used extraction kits. The DNA extraction was executed following the DNeasy Power Soil Pro kit (ID: 4701, Qiagen). The RNeasy Power Biofilm kit (ID: 25000–50, Qiagen) was used for the RNA extraction. The concentration of the DNA and RNA were measured using DeNovix dsDNA Broad Range Kit (Thermo Fisher, #Q3284) and Qubit RNA Broad Range Assay Kit (Thermo Fisher, Invitrogen #Q32852), respectively. DNA samples were taken over the whole reactor height on days 6, 17, and 52, while RNA extracts were retrieved over the whole time, 49 cm from the reactor inlet, and once over the whole height on day 20 (Fig. 1B). The chosen samples aimed to cover temporal and spatial variances and changes of the microbial community with a focus on fungi, while getting insight into all present organisms, as well as the active biomass.

### RNA preparation and transcription

The extracted RNA was tested for DNA contamination using a PCR and gel electrophoresis. The PCR was performed in 25 μL reactions with 12,5 μL GoTaq Green Master Mix (Promega, #M7123), 5 pmol of each primer, and 1 μL template. We ran an initial denaturation of 2 min at 95 °C, then 25 cycles at 95 °C for 15 s, 60 °C for 30 s, and 68 °C for 2 min. An 8% agarose gel was used for the electrophoresis. When DNA contaminations were present in the RNA extracts, we cleaned them using the TRUBO DNA-free kit (Thermo Fisher, Invitrogen #AM1907). The GoScript Reverse Transcription System (Promega, TM316) was used for reverse transcription of RNA into cDNA.

### Quantitative real‐time PCR (qPCR)

DNA Samples over the whole reactor height on days 6, 17, and 52, as well as cDNA on day 20, were used to quantify *Rozellomycota* and bacterial ribosomal copy numbers. The approximation of a general bacterial abundance was based on the 16S rRNA gene from both the DNA and the cDNA using the primer pair forward 337 (5’-GACTCCTACGGGAGGCAGCAG-3’) and reverse 518 (5’-GTATTACCGCGGCTGCTGG-3’)^21^. Each qPCR was performed with two technical and five biological replicates in 21 μL reactions with 10 μL of GoTaq qPCR Master Mix (Promega, #A6001), 2.5 pmol of each primer, and 1 μL template. We ran an activation of 2 min at 95 °C, then 40 cycles at 95 °C for 15 s and 60 °C for 60 s. To profile the fungal community, a new primer set targeting *Rozellomycota* was used, consisting of 5.8S30fwd (5’-CTTTTAACAATGGATCTCTAGGCTC-3’) and 5.8S130rev (5’-GTTCAAAGATTAGATGACTCACAGA-3’)^20^. The qPCR was performed in a 20 μL qPCR assay with 10 μL SSOAdvanced Universal SYBR Green Supermix (Bio‐Rad Laboratories, Inc.), 5 pmol of each primer, and 1 μL of template. The initial activation/denaturation took place for 4 min at 95 °C, followed by 35 cycles of a two‐step PCR with a denaturation step at 95 °C for 10 s and the annealing step at 62 °C for 20 s. Both RT-qPCRs were carried out in duplicate on a CFX96 thermal cycler (Bio‐Rad Laboratories, Inc.).

### DNA and cDNA amplicon sequencing and data processing

Spatially taken DNA samples (taken from all heights on day 6, 17, and 52), and temporal cDNA samples from 49 cm for all time points (n = 10) with five replicates respectively were sequenced by amplicon sequencing. The primer set 341F (5’-CCTACGGGNGGCWGCAG-3’) and 785R (5’-GACTACHVGGGTATCTAATCC-3’) was used, targeting the V3 and V4 region of the 16S rRNA to generate prokaryotic, bacterial, and archaeal data^22^. To analyze the eukaryotic community composition, the V4 region of the 18S rRNA was targeted using TAReuk454FWD1 (5’-CCAGCASCYGCGGTAATTCC-3’) and TAReukREV3 (5’-ACTTTCGTTCTTGATYRA-3’)^23^. An Illumina MiSeq system with V3 chemistry was used to sequence the amplicons, and sequencing data was received as consensus FASTQ sequences after the overlap-combination of forward and reverse reads. The sequencing was done externally with LGC Genomics GmbH and at the ZIEL Institute for Food & Health Core Facility Mikrobiom (Technical University of Munich).

FASTQ files were imported into R v4.3.3 for data processing following the DADA2v1.28.0^24^ pipeline. Following the pipeline, ASV tables were achieved after quality trimming, denoising, error correction, paired-end read merging, chimaera removal, and dereplication steps. Taxonomic assignment of ASVs was performed using the RDP Naive Bayesian Classifier algorithm^25^ against the SILVA SSU database v132^26^. For further community analysis, the ASVs table, taxonomy table, and sample metadata were imported into Phyloseq v1.44.0^27^, applying Biostrings v2.68.1^28^.

### Statistical and correlation analysis

All analyses performed comprise the data of all five replicated reactors. Data analysis was done with R v4.3.3. Shannon index was calculated and visualized for the alpha diversity analysis using the R packages Phyloseq v 1.44.0^27^ and ggplot2 v 3.4.3^29^. Non-metric multidimensional scaling (NMDS) analysis was performed by the R package vegan v 2.6-4^30^ based on monotone regression and primary treatment of ties, including significant envfit results. Permutational Multivariate Analysis of Variance (PERMANOVA) analysis of the amplicon sequencing results and sampling time and position were performed using vegans (v 2.6-4^30^) adonis2 function. The R packages Phyloseq v 1.44.0^31^ and ggplot2 v 3.4.3^29^ were used for the relative abundance analysis and visualization. The Spearman correlations of diversity and average removal rates were performed using the R package ClusterNet, applying the cutoff of r > 0.6 and a *p*-value < 0.05. The correlations were not corrected for multiple comparisons.

## Results

### DHS reactors and process performance

A distinct change in the polyurethane filling could be seen during the eight-week experiments. Starting as clean white sponges, a colouration towards light and dark brown (Fig. 1A), and the accumulation of sludge-like substances over the course of the experiment could be observed. At the top of the filling material, filtration processes cause a build-up of suspended solids. Further down the system, microbial growth with concomitant biofilm production is seemingly the main driver of the change.

**Fig. 2:**
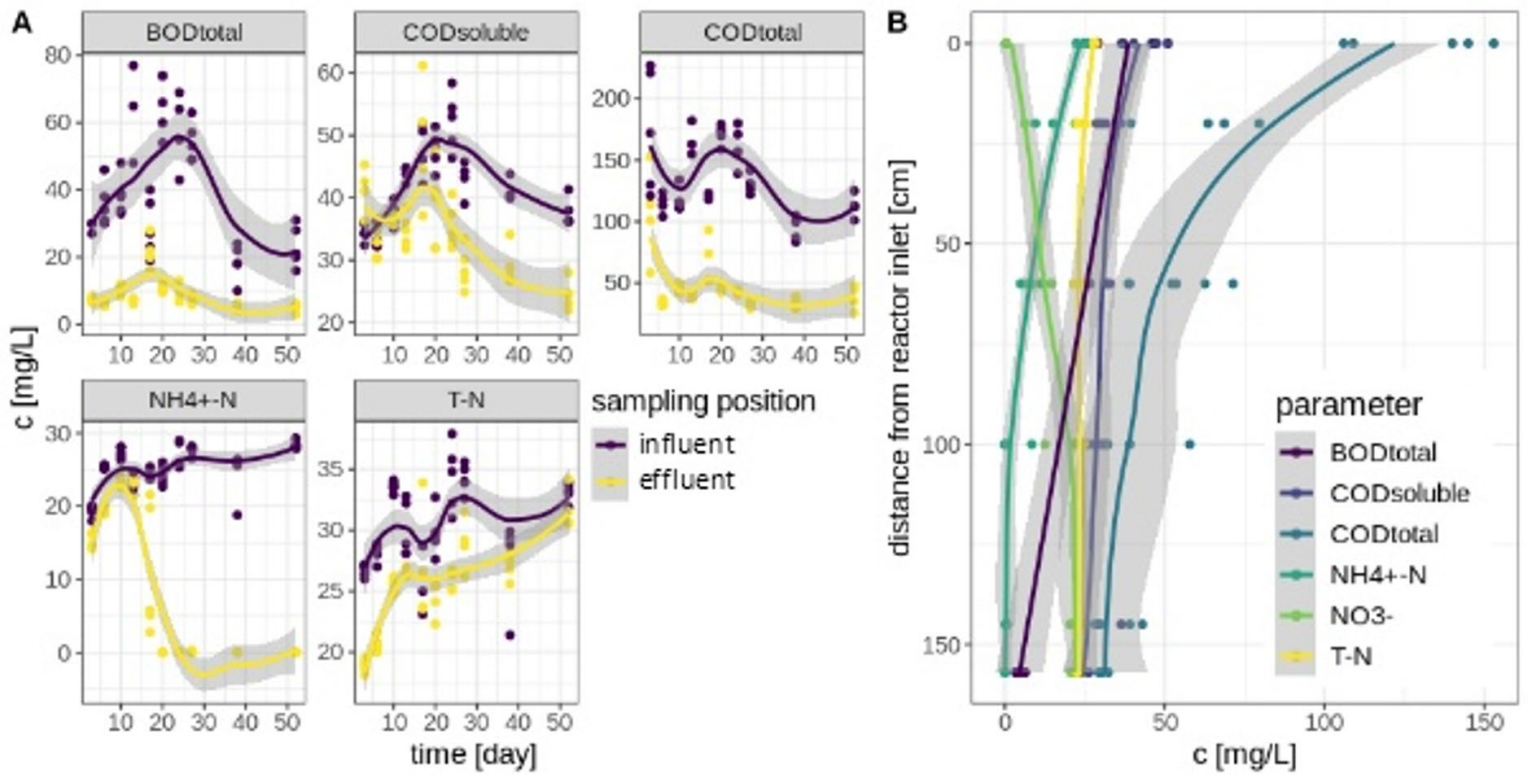
(**A**) Concentration of measured water parameters (BOD, COD [soluble and total], ammonium, and total nitrogen, in five biological replicates) in mg/L of influent (violet) and effluent (yellow) of the five reactor systems over the time of the experiments(water sampling days 3, 6, 10, 13, 17, 20, 24, 27, 38, and 52, in five biological replicates). (**B**) Concentration of measured water parameters (BOD [violet], soluble COD [blue], total COD [teal], ammonium [dark green], nitrate [light green], and total nitrogen [yellow]) in mg/L on day 38 of process time over the height of the reactors (sampling position 20, 60, 100, and 145 cm from inlet).

The removal efficiency of all observed parameters also changed throughout the experiment (Fig. 2A, Supplementary Table 1). The ammonium reduction increased significantly over the first 17 days (*p* < 0.01), indicating the accumulation of a nitrifier community, leading to a high nitrification rate of nearly 100% from day 38 onwards. A relatively low and inconsistent total nitrogen reduction suggests a lack of and instability of denitrifying processes (26.61 ± 2.66% to 6.53 ± 3.82%). Over the whole runtime of the reactors, average BOD_total_ and COD_total_ removal displayed efficiencies of 73.88 ± 13.64% and 67.54 ± 8.85%, respectively, with a reduced BOD removal towards the end of the experiment (Supplementary Table 1). The reduction of COD_soluble_, on the other hand, significantly increased over the time of the experiment (*p* < 0.01), starting at 3.41 ± 8.64% and reaching up to 45.97 ± 6.28% on day 38. Variability of influent parameters (Fig. 2A) was mostly caused by natural fluctuations of the water quality of the municipal wastewater treatment plant (e.g., the BOD and COD influent on days 23 and 20) that was used as a feed source in combination with a manual sequencing batch operation mode.

Over the height of the reactors, the ammonium and COD (total and soluble) concentrations decreased while the nitrate concentration increased (Fig. 2B). Most COD removal is seen in the upper third of the systems, where most suspended solids are retained. Looking at the inverse relationship between ammonium and nitrate, nitrification mainly took place in the upper two-thirds of the systems. The denitrification rate appeared to be low, as seen in the low total nitrogen removal, leading to the assumption that no active denitrifying microbial community was present in our DHS setup.

### Microbial communities over the reactor height

**Fig. 3:**
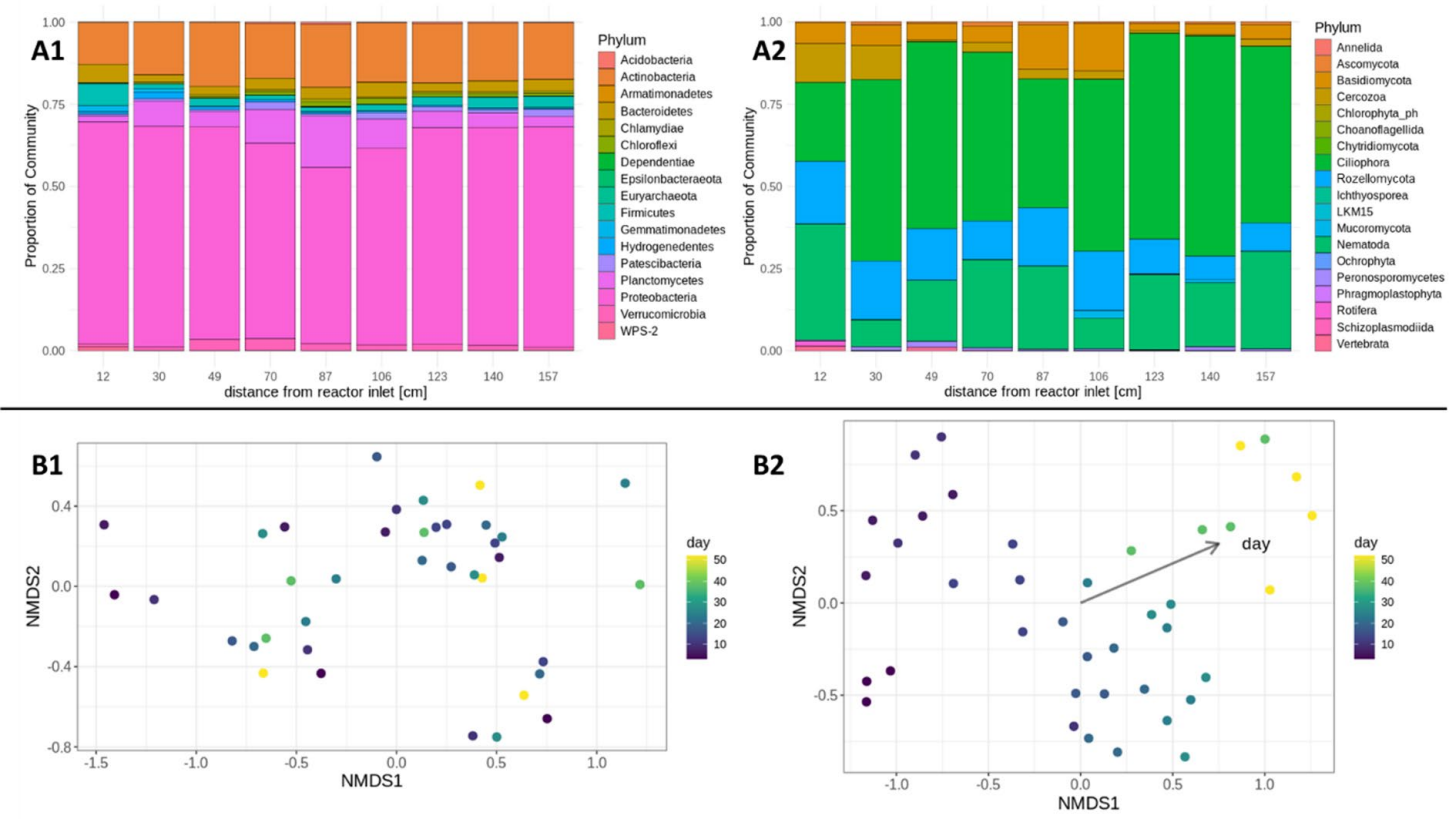
(**A**) Relative abundance (including five biological replicates per position) of prokaryotic 16S DNA (**A1**) and eukaryotic 18S DNA (**A2**) taxa per reactor heights (12, 30, 49, 70, 87, 106, 123, 140, and 157 cm distance from reactor inlet) on day 52. (**B**) Non-metric Multi-dimensional Scaling (NMDS) plot (including five biological replicates per day) of 16S (**B1**) and 18S (**B2**) cDNA community analysis over the whole experiment run time (sampling days 3, 6, 10, 13, 17, 20, 24, 27, 38, and 52) at 49 cm of reactor height. Stress = 0.1204 and 0.1680, respectively. Envfit correlation with time for 18S: r^2^ = 0.896, *p* < 0.001.

After confirming stable DHS system performance, this study focuses on the microbial community within the reactors. Water parameter profiles along the reactor height motivated an investigation of corresponding microbial gradients. To assess taxonomic composition, particularly the niche of *Rozellomycota*, we analysed DNA-based 16S and 18S amplicons at the end of the experiment (day 52) across the full reactor height. Significant but small effects in the bacterial and eukaryotic community composition over the height of the reactors could be seen (adonis, R^2^ = 0.053, *p* < 0.001; R^2^ = 0.042, *p* < 0.05, respectively) (Fig. 3A1 and A2, respectively, Supplementary Fig. 2). The distribution of dominant prokaryotic sequencing variants appeared to be rather constant, showing a very weak specificity for the reactor’s identity (adonis, R^2^ = 0.053, *p* < 0.001). *Proteobacteria* ASVs were dominant in 16S DNA and cDNA samples (73.99 ± 11.68%; 61.10 ± 11.71%, respectively), followed by *Actinobacteria* (12.51 ± 6.04%; 8.54 ± 4.77%, respectively) and *Bacteroidetes* (2.79 ± 1.18%; 6.01 ± 2.17%, respectively). The only archaeal lineage belonged to *Euryarchaeota* at low abundances. Analysis of the relative eukaryotic community revealed *Ciliophora* (54.38%) and *Nematoda* (21.68%) sequences as the most abundant biofilm community members, followed by *Rozellomycota* (12.86%). rozellomycotean ASVs were found over the whole height of the reactor and appeared as the dominant microeukaryote (Supplementary Table 2). No significant correlations between the eukaryotic community diversity and sample height could be observed. The alpha diversity of prokaryotes showed significant height dependancies (Spearman r = 0.436, *p* < 0.001) with a trend towards an increased diversity at the lower half of the reactor systems(Supplementary Table 3). With Shannon indices between 3.65 and 6.38, prokaryotes were, as expected, more diverse than eukaryotes, with indices between 2.32 and 3.11.

### Temporal succession of Bacteria and Eukaryotes

Examining the progressive succession over time gives further insight into the niche formation. RNA-based cDNA data representing the living microorganisms from 16 to 18S were analysed over the entire run time of 52 days at 49 cm (upper third) from the reactor inlet to enlighten the progressive succession of the active microbial community (Fig. 3B, Supplementary Fig. 3). Looking into the alpha diversity of the active microbial community, the 16S and 18S cDNA diversity showed no significant changes over time (Supplementary Fig. 1a). Still, the 18S cDNA diversity showed a trend towards a higher diversity between days 13 and 27 of the reactor run time (Supplementary Fig. 1b). This points to the expected lag of growth in eukaryotic organisms and the longer time needed to find fitting niches. Also, the following decrease in diversity could show a temporal selection.

NMDS ordinations were used to visualize the sample dissimilarity of the active microbial community composition over the time of the experiment. In the cDNA data of eukaryotes (Fig. 3B2), significant strong correlations between the community structure and the sampling time (envfit, r2 = 0.896, *p* < 0.001) as well as significant correlations across groups defined by the sampling time can be seen (adonis, R^2^ = 0.201, *p* < 0.001). This temporal gradient was absent in the prokaryotic community composition (Fig. 3B1), underlining a quick community assembly.

### Prominence of *Rozellomycota*

Similar to the DNA-based data, *Rozellomycota* are the second most abundant group (9.57%) after *Ciliophora* (80,91%) in the cDNA data (Supplementary Table 2). Rozellomycotean cDNA was present starting on the second sampling day, day 6 (Supplementary Fig. 3). Considering solely the *Rozellomycota* community composition, a significant correlation between ASVs and time (adonis, R^2^ = 0.171, *p* < 0.001), ammonium removal (adonis, R^2^ = 0.068, *p* < 0.05), and *Rozellomycota* copy numbers (adonis, R^2^ = 0.171, *p* < 0.05) was observed (Supplementary Fig. 4B).

**Fig. 4:**
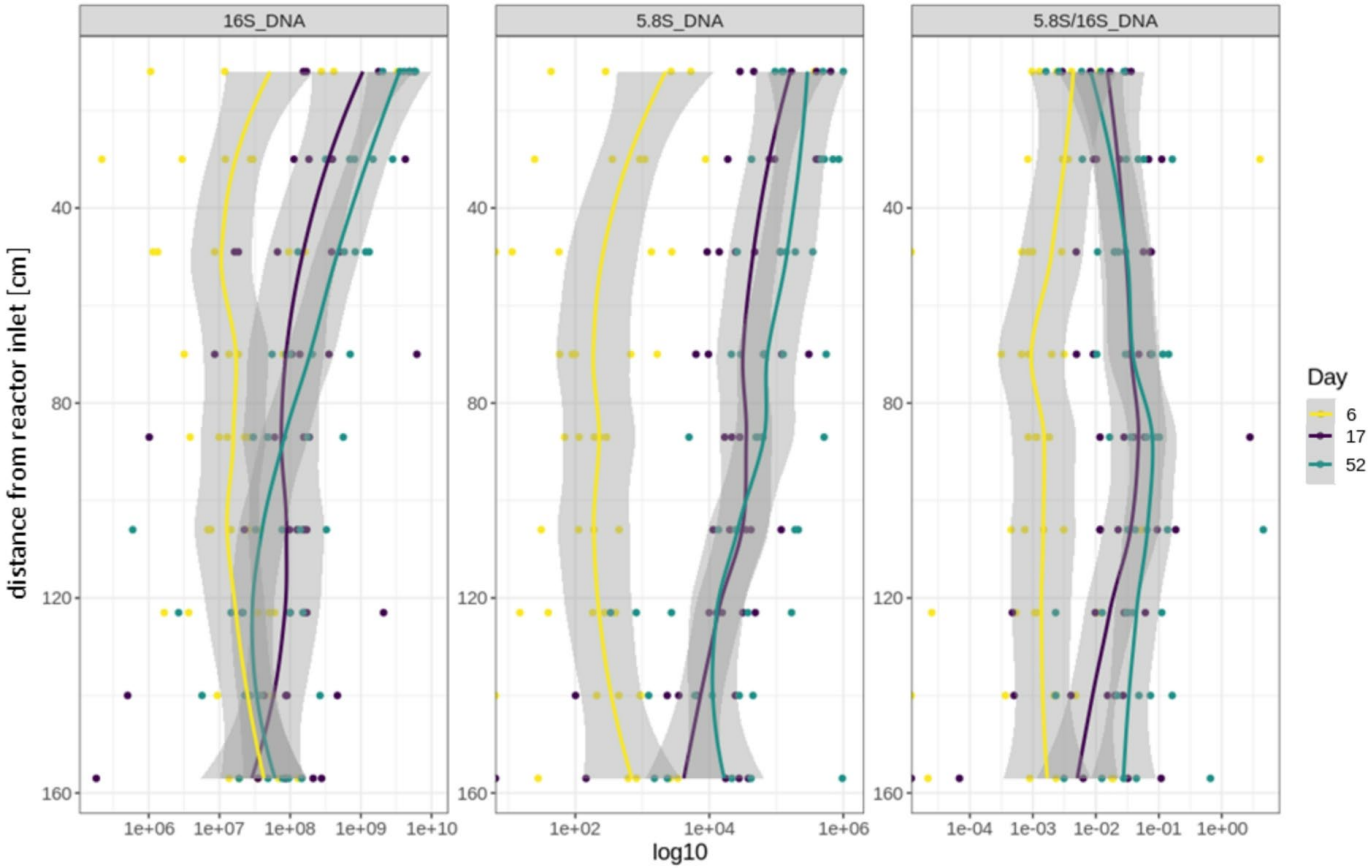
DNA qPCR results (five biological and two technical replicates)- 16S copies (left), *Rozellomycota* 5.8S copies (middle), and 5.8S/16S ratio (right) in DHS reactor systems over height and time (day 6 [yellow], day 17 [violet], day 52 [teal]).

16S and 5.8S-*Rozellomycota* qPCR were used to get quantitative insight into the progressive succession of the microbial community in the systems over the course of the experiment and reactor height. A moderate to strong Spearman correlation between bacterial and *Rozellomycota* copy numbers could be seen in the DNA data (r = 0.745, *p* < 0.001), similar to the results of the cDNA based copy numbers (r = 0.672, *p* < 0.001). *Rozellomycota* (5.8S results) showed abundances between 10^1^ and 10^6^ copies per μL in all analysed samples, and their maximum abundance was delayed at day 6 in comparison to bacteria (16S results), reaching a constant copy number (abundance) only later at day 17 and 52 (Fig. 4). This supports the assumption that the fungal growth rate is smaller than the bacterial growth rate and/or that the niche formation of fungi takes some time. A weak Spearman correlation (r = − 0.355, *p* < 0.001) with the reactor height was observed in the bacterial DNA (16S), with the bacteria being more abundant at the top of the systems, especially on days 17 and 52. A higher nutrient concentration due to retention processes on top likely causes the slight decrease in biomass with height. The *Rozellomycota* qPCR results (5.8S) show a comparable decrease with height (Spearman r = − 0.355, *p* < 0.001). However, in comparison with 16S, the *Rozellomycota* were slightly more pronounced in the middle to lower part (70–123 cm) of the systems in relation to bacteria (5.8S/16S, Fig. 4). This could be due to host organisms being present in this part of the reactor or a corresponding niche related to the water chemistry. This is also supported by the cDNA data (Supplementary Fig. 5).

### Niche of *Rozellomycota* in the DHS systems

Investigating the fungal phyla detected in the amplicon dataset, we see that *Rozellomycota* are the most abundant fungi. 49 *Rozellomycota* ASVs were found in the cDNA analysis. The three most abundant of those ASVs accounted for 4.95 ± 4.41%, 1.83 ± 1.66%, and 0.97 ± 1.49% of all eukaryotic organisms in the samples (Supplementary Table 4). Analysing the DNA, 53 *Rozellomycota* ASVs, with the three highest abundant ones presenting 5.62 ± 3.11%, 3.18 ± 1.69%, and 0.96 ± 1.02% of all eukaryotes. The abundance and diversity of *Rozellomycota* ASVs in both DNA and cDNA sequencing results underline the hypothesis that they play an essential role in the biofilm microbial community of the reactor. Simultaneously, it points to them fulfilling a crucial niche in the degradation of nutrients in the system at hand over the whole experiment time.

The influence of *Rozellomycota* on system performance and metabolic cycles was further evaluated by Spearman correlations between quantitative and qualitative community analysis results (qPCR results, ASVs, respectively) and removal efficiencies. No significant correlations could be found in the cDNA data (Supplementary Fig. 6). In the DNA-based analysis, *Rozellomycota* and bacterial DNA copy numbers showed a significant negative Spearman correlation with total nitrogen decrease (r = − 0.73, *p* < 0.01; r = -0.85, *p* < 0.001). The normalised 5.8S qPCR results showed no significant correlations to any water parameter. Inspecting the ASV taxonomic groups, only LKM15 showed a light negative Spearman correlation (r = − 0.34, *p* < 0.05) with ammonium decrease (Supplementary Fig. 7).

*Rozellomycota* ASVs and bacterial ASVs showed several moderate to strong correlations. In the DNA results, six *Rozellomycota* ASVs and two LKM15 ASVs were correlated with predominantly *Proteobacteria* ASVs belonging mostly to *Alpha*- and *Gammaproteobacteria* (Supplementary Fig. 8B). Seven additional *Rozellomycota* cDNA ASVs correlated with *Proteobacteria*, *Actinobacteria*, and *Bacteroidetes* ASVs (Supplementary Fig. 8A). Furthermore, all correlations with *Rozellomycota* ASVs were positive. Within the eukaryote dataset, single *Rozellomycota* ASVs only correlated with *Ciliophora* ASVs, partly positive and partly negative. Also, some correlations of *Rozellomycota* ASVs to unclassified ASVs were present (Supplementary Tables 5 and 6).

## Discussion

With a near 100% ammonia removal, the nitrification process seen is supported by literature on DHS reactors, although the proposed denitrification was missing^7,32,33^. Most likely, no stable anoxic zone developed inside the sponges, not providing the environment for the typical facultative anaerobic denitrifiers^34^. Total BOD and COD removal efficiencies (73.88 ± 13.64%, 67.54 ± 8.85%, respectively) were lower than full-scale engineered DHS reactors but still within the range of experimental facilities^14,33,35^. The obtained qPCR data indicate that prokaryotes constitute the predominant fraction of the samples, consistent with their established role as key drivers of biofilm formation in WWTPs^36^. The analysis of eukaryotic ASVs revealed *Ciliophora* to be the most abundant (54.4% and 80.9% in DNA and cDNA results, respectively), followed by *Nematoda* (21.7% and 4.5%), or *Rozellomycota* (12.9% and 9.6%), which represent typically highly abundant genera in previous DHS studies^7,37^. Those high abundances and fungal dominanceof *Rozellomycota* in all samples supported the results of past studies, like Miyaoka et al.^7^ and Watari et al.^38^. In this study, we have investigated the dynamics of biofilm establishment with wastewater over time and a height gradient. While our samples did not investigate the wastewater community itself, our study focuses on the colonization of biofilm carriers itself across the first two months (day 3–52). Apparently, our study is thus not considering the initial inoculation process by the wastewater community during the first two days, which may be crucial for fast growing bacteria, but is rather focusing on how the Eukaryotes and the *Rozellomycota* within are establishing their niche slowly over time.The data show *Rozellomycota* ASVs in all cDNA samples, except for sampling day 3, representing the first analyzed samples. Although the study does not include sequencing of the influent, this leads to the conclusion that the main *Rozellomycota* community developed inside the DHS carriers and was only introduced as inoculum. More importantly, the increase of *Rozellomycota* abundances from the first settlement at day 6 to day 17 by two orders of magnitude, cannot be explained by immigration.

Previous studies addressed the potential effects of *Rozellomycota* on food-web dynamics. Gleason et al.^39^ identified the species *Rozella* as a part of the carbon transfer from primary (hosts) to tertiary consumers (grazing zooplankton)^40,41^ as well as regulators of the primary consumer population, and, therefore, the inhibition of detritus decomposition^42^. The ecological potential in the study at hand was analysed using correlation analysis. Although no distinct and reliably replicated correlations of *Rozellomycota* AVSs with eukaryotic organisms were observed, various interactions with *Ciliophora* were present in the data. This could point, on the one hand, to the known and well-described parasitic lifestyle of the microfungi with *Ciliophora* themselves, or *Ciliophora* infecting fungi^1,11,43^. It is, on the other hand, also proposed that grazing zooplankters, like *Ciliophora*, are able to feed on zoospores up to 5 µm^40,44^. Zoospores produced by *Rozellomycota* fall below this threshold and provide a valuable food source with their high fatty acid and sterol content^40,44^. Therefore, the literature provides the foundation to explain positive and negative correlations between *Rozellomycota* and *Ciliophora*, as seen in the data. The analysis of prokaryotic sequences revealed correlations with several bacteria ASVs, mostly *Proteobacteria*, *Actinobacteria*, and *Bacteroidetes*, which also showed the highest bacterial abundances. The important role of those phyla in nutrient degradation in wastewater treatment^45–47^ and, therefore, in the reactor systems and the corresponding high abundance can explain the clear correlations in the results.

The sequencing analysis gives insight into the qualitative composition of the microbial community. Since the sequence representation is always affected by PCR biases^48^, a quantitative method, like qPCR, needs to be considered to get reliable insight into the dominance of the *Rozellomycota.* The correlation of *Rozellomycota* (including the clade LKM15) abundances and changes in water parameters inside the system was tested using ASV and qPCR results, revealing negative correlations with ammonium and nitrogen concentration decrease, respectively. Studies have already suggested the role of aquatic fungi in the nitrogen cycle in marine ecosystems^49^. They were, however, positively associated with denitrification, nitrite and nitrate reduction, and ammonia formation in anoxic zones^50–52^.

Those mixed correlations of *Rozellomycota* with *Ciliophora*, and bacteria, and negative correlations of the abundances with ammonium and nitrogen concentrations may not be causal and require future research to further investigate a putative causality. They could point to their role within the microbial community in WWTP-like systems and ultimately help fidentify optimization possibilities for wastewater treatment. *Rozellomycota* could be considered an essential factor in regulating the primary consumer population. Still, further research is needed to validate the results, add further knowledge, and overcome the limitations of this study. The question of the active role of *Rozellomycota* requires additional experiments to be answered. Since they appear to be dominant, stable isotope labeling of the fed carbon sources coupled with DNA or cDNA sequencing could also elucidate the carbon flux and active degradation role of the organisms of interest. These steps should further lead to sampling full-scale WWTPs to investigate the *Rozellomycota* in the more complex natural habitat and integrate imaging methods like FISH to validate possible host organisms.

## Conclusion

The results of the study gave insight into the dynamics and establishment of *Rozellomycota* inside the DHS systems as WWTP model systems over different parameters. Overall, the sequencing results revealed the *Rozellomycota* as the third-most abundant eukaryote with 53 ASVs within DNA-based results and the second most abundant within the active eukaryotic biomass, showing 49 ASVs. Overall, they were the most abundant and dominant fungi and microeukaryotes in all tested samples. Correlation analysis revealed correlations between *Rozellomycota, Ciliophora,* and bacterial ASVs, pointing to possible crossfeeding mechanisms and parasitic lifestyles. Furthermore, a negative correlation with nitrogen species but not with carbon removal could be observed.

Overall, the findings underline the hypotheses of *Rozellomycota* being highly diverse and dominant in WWTP-like systems. Furthermore, the expected delayed growth can be supported, as well as the presence of the fungal phylum in the whole system. Our results point to an active function of the organisms in the wastewater biofilm.

## Supplementary Information

Below is the link to the electronic supplementary material.

Supplementary Material 1

Supplementary Material 2

## Data Availability

All data generated or analysed during this study are included in this published article (and its Supplementary Information files). The sequencing datasets generated during and/or analysed during the current study are available in the European Nucleotide Archive (ENA) repository, https://www.ebi.ac.uk/ena/browser/view/PRJEB90848.
