# Supplementary material for: Niche formation of Rozellomycota in wastewater treatment model reactors
Source: Sci Rep. 2025 Oct 15;15:36105. doi: 10.1038/s41598-025-23542-6 (PMC12528753; doi:10.1038/s41598-025-23542-6)
Supplement: Supplementary file 2 — Supplementary Material 2 [file 41598_2025_23542_MOESM2_ESM.docx]

**Niche Formation of *Rozellomycota* in Wastewater Treatment Model Reactors**

Katrin Stüer-Patowsky, Eva Gega, Lijia Cao, Christian Wurzbacher

Supplementary Table 1: Removal efficiency, calculated as E(%) = (c_in_ − c_out_)/c_in_ x 100, of water parameters (BOD, COD, NH_4_^+^-N and T-N) over 52 days of DHS system runtime.

|  | Removal efficiency [%] | | | | |
| --- | --- | --- | --- | --- | --- |
| day | BOD_total_ | COD_total_ | COD_soluble_ | NH_4_^+^-N | T-N |
| 6 | 83.26 ± 2.94 | 73.09 ± 4.85 | 3.41 ± 8.64 | 23.33 ± 0.90 | 26.61 ± 2.66 |
| 17 | 39.77 ± 6.82 | 50.92 ± 8.33 | -8.28 ± 8.46 | 54.87 ± 31.71 | 5.79 ± 15.94 |
| 20 | 83.79 ± 1.89 | 74.62 ± 1.30 | 19.23 ± 10.37 | 85.21 ± 23.47 | 17.05 ± 5.90 |
| 38 | 87.54 ± 3.43 | 77.03 ± 2.1 | 45.97 ± 6.28 | 99.81 ± 0.04 | 18.52 ± 1.81 |
| 52 | 75.04 ± 7.41 | 62.04 ± 9.82 | 34.79 ± 5.65 | 99.81 ± 0.04 | 6.53 ± 3.82 |

Supplementary Table 2: Percentage of taxa abundance in all 18S DNA and cDNA samples.

|  | 18S DNA [%] | | 18S cDNA [%] | |
| --- | --- | --- | --- | --- |
| *Taxa* | *mean* | *dev* | *mean* | *dev* |
| *Arthropoda* | 0.08 | 0.00 | 0.01 | 0.00 |
| *Ascomycota* | 0.64 | 0.01 | 0.19 | 0.01 |
| *Basidiomycota* | 5.81 | 0.17 | 1.00 | 0.06 |
| *Cercozoa* | 2.82 | 0.12 | 0.16 | 0.01 |
| *Chlorophyta_ph* | 0.03 | 0.00 | 0.01 | 0.00 |
| *Choanoflagellida* | 0.05 | 0.00 | 0.08 | 0.01 |
| *Chytridiomycota* | 0.10 | 0.01 | 0.00 | 0.00 |
| *Ciliophora* | 54.38 | 0.90 | 80.91 | 1.79 |
| *Rozellomycota* | 12.86 | 0.18 | 9.57 | 0.42 |
| *Ichthyosporea* | 0.01 | 0.00 | 0.02 | 0.00 |
| LKM15 | 0.03 | 0.03 | 0.08 | 0.01 |
| *Mucoromycota* | 0.30 | 0.33 | 0.09 | 0.01 |
| *Nematoda* | 21.68 | 0.61 | 4.48 | 0.46 |
| *Ochrophyta* | 0.07 | 0.00 | 2.66 | 0.22 |
| *Peronosporomycetes* | 0.66 | 0.03 | 0.14 | 0.01 |
| *Phragmoplastophyta* | 0.09 | 0.00 | 0.20 | 0.01 |
| *Protalveolata* | 0.01 | 0.00 | 0.04 | 0.00 |
| *Rotifera* | 0.13 | 0.01 | 0 | 0.00 |
| *Schizoplasmodiida* | 0.02 | 0.00 | 0.28 | 0.03 |
| *Vertebrata* | 0.22 | 0.00 | 0.05 | 0.00 |

Supplementary Table 3: Alpha diversity of 16S and 18S DNA per height and reactor taken after 52 days of operation.

|  |  | Shannon | | | |
| --- | --- | --- | --- | --- | --- |
|  |  | 16S | | 18S | |
| height | day | mean | dev | mean | dev |
| 12 | 52 | 5.42 | 1.54 | 3.11 | 0.39 |
| 30 | 52 | 4.26 | 1.27 | 2.61 | 0.44 |
| 49 | 52 | 3.65 | 1.74 | 2.69 | 0.76 |
| 70 | 52 | 5.59 | 1.05 | 2.60 | 0.44 |
| 87 | 52 | 5.49 | 1.52 | 2.35 | 0.36 |
| 106 | 52 | 5.52 | 1.33 | 2.52 | 0.42 |
| 123 | 52 | 5.47 | 1.47 | 2.29 | 0.71 |
| 140 | 52 | 6.25 | 1.80 | 2.32 | 0.56 |
| 157 | 52 | 6.38 | 1.76 | 2.47 | 0.35 |


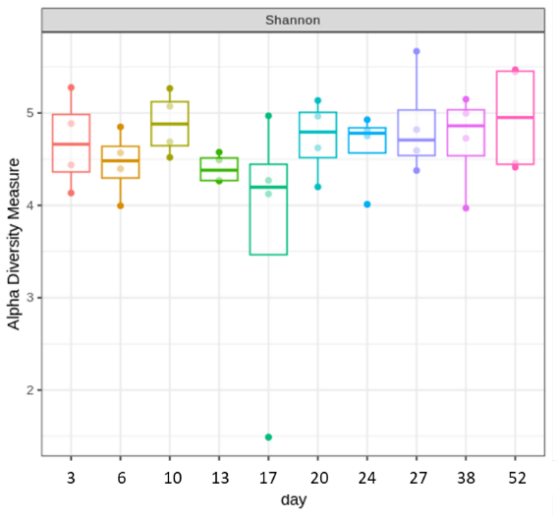

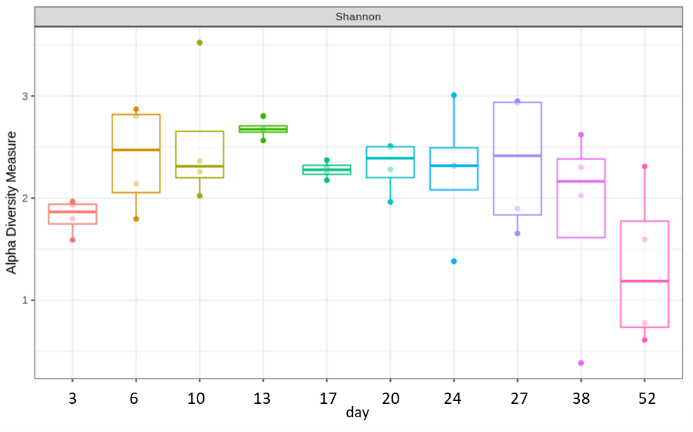


(b)

(a)

Supplementary Figure 1: Alpha diversity of (a) 16S cDNA and (b) 18S cDNA per day taken at 49 cm of reactor height.


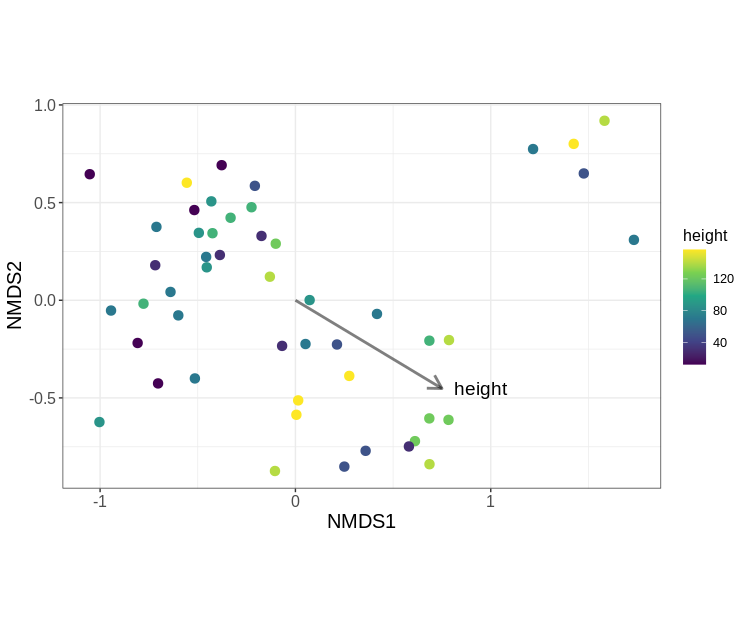

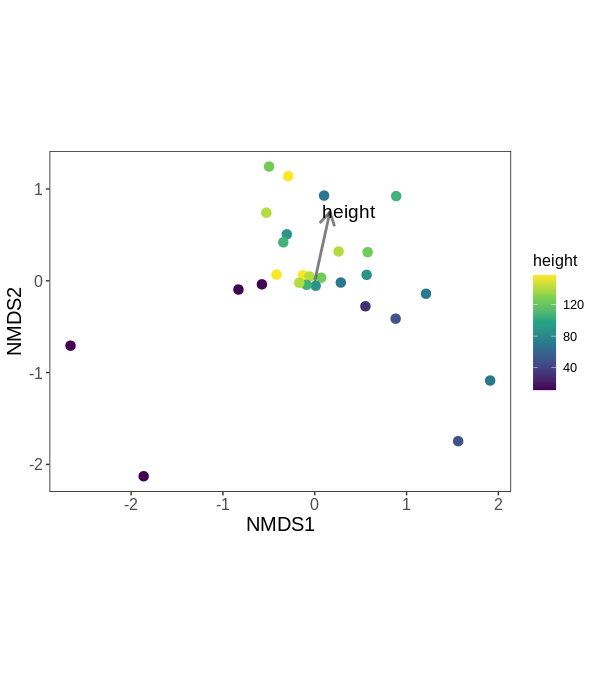


Supplementary Figure 2: Non-metric Multi-dimensional Scaling (NMDS) plot of (a) 18S DNA (Dependency on sampling height envfit results: r^2^ = 0.130, p < 0.05) and (b) 16S DNA (Dependency on sampling height envfit results: r^2^ = 0.330, p < 0.01) community analysis on different reactor heights. Stress=0.1488/0.1215.


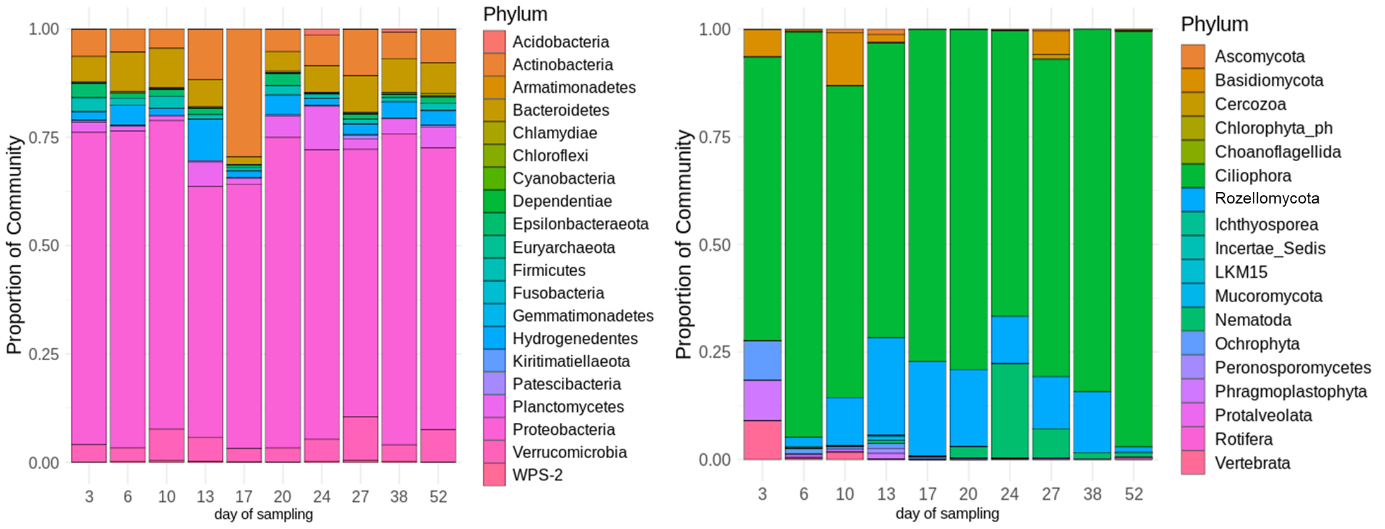


(b)

(a)

Supplementary Figure 3: Relative abundance (including five biological replicates per position) of prokaryotic 16S cDNA (a) and eukaryotic 18S cDNA (b) taxa per sampling day (3, 6, 10, 13, 17, 20, 24, 27, 38, and 52) at 49 cm of reactor height.


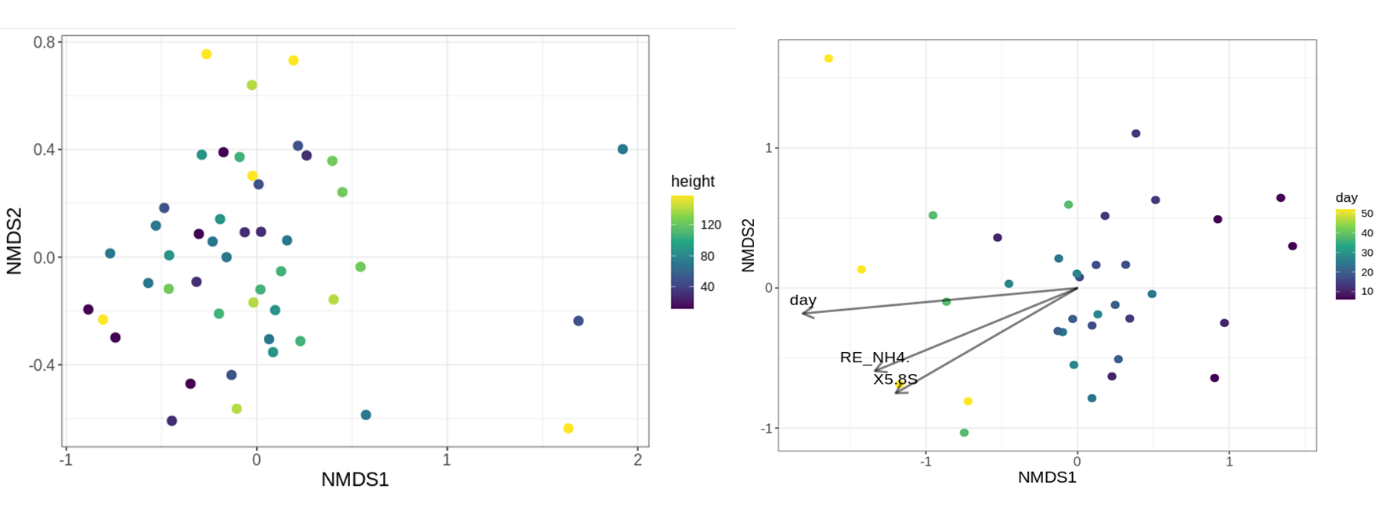


(b)

(a)

Supplementary Figure 4: Non-metric Multi-dimensional Scaling (NMDS) plot of (a) Rozellomycota 18S DNA community analysis on different reactor heights and (b) Rozellomycota 18S cDNA (Dependency on sampling day/NH4+-removal envfit results: r^2^ = 0.890/0.573, p < 0.001/0.01) on different days taken at 49 cm of reactor height. Stress=0.1969/0.1947.

Supplementary Table 4: Percentage of the 13 most abundant Rozellomycota of all ASVs (n) found in 18S cDNA and DNA samples.

|  | *Percentage of eukaryotes [%]* | |  |  | *Percentage of eukaryotes [%]* | |
| --- | --- | --- | --- | --- | --- | --- |
| *cDNA ASVs* | *mean* | *dev* |  | *DNA ASVs* | *mean* | *dev* |
| ASV_5 | 4.95 | 4.41 |  | ASV_7 | 5.62 | 3.11 |
| ASV_14 | 1.83 | 1.66 |  | ASV_10 | 3.18 | 1.69 |
| ASV_18 | 0.97 | 1.49 |  | ASV_23 | 0.96 | 1.02 |
| ASV_38 | 0.32 | 0.32 |  | ASV_31 | 0.47 | 0.35 |
| ASV_47 | 0.18 | 0.26 |  | ASV_36 | 0.16 | 0.26 |
| ASV_86 | 0.04 | 0.07 |  | ASV_66 | 0.11 | 0.14 |
| ASV_88 | 0.04 | 0.06 |  | ASV_69 | 0.13 | 0.22 |
| ASV_103 | 0.04 | 0.07 |  | ASV_76 | 0.07 | 0.09 |
| ASV_105 | 0.04 | 0.05 |  | ASV_106 | 0.04 | 0.03 |
| ASV_115 | 0.04 | 0.06 |  | ASV_117 | 0.03 | 0.04 |
| ASV_142 | 0.02 | 0.03 |  | ASV_135 | 0.02 | 0.04 |
| ASV_152 | 0.03 | 0.04 |  | ASV_142 | 0.02 | 0.03 |
| ASV_154 | 0.03 | 0.07 |  | ASV_147 | 0.01 | 0.02 |
| … | … | … |  | … | … | … |
| n | 49 | |  | n | 53 | |

*
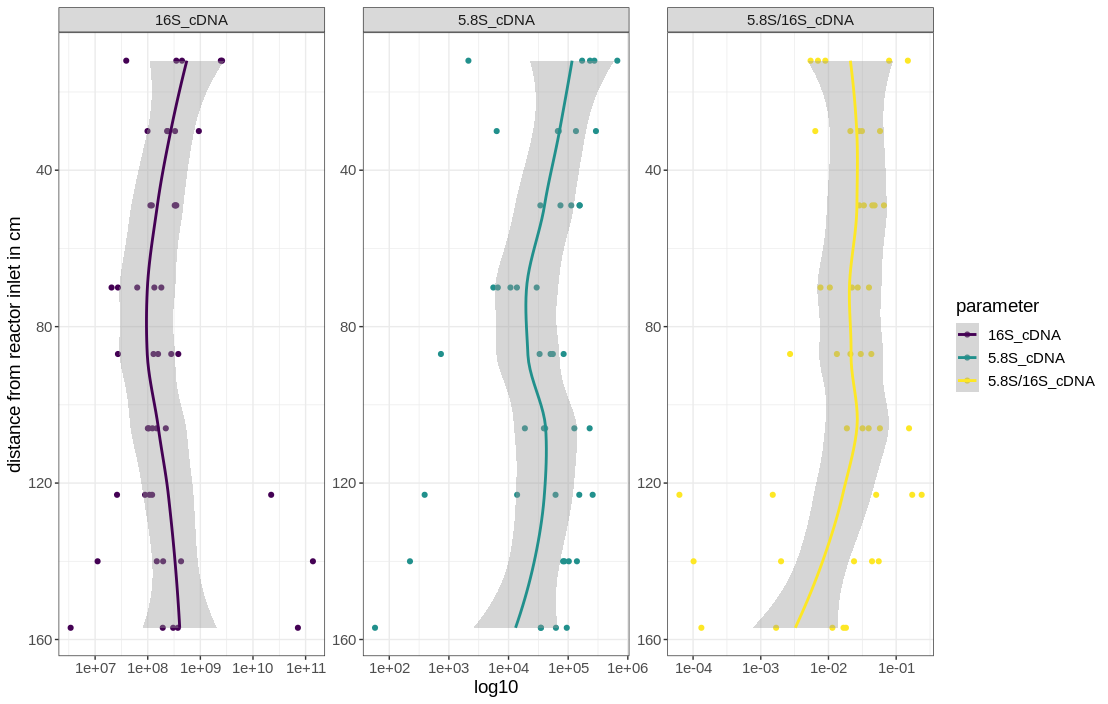
*

Supplementary Figure 5: 5.8S cDNA qPCR results over the reactors' height on day 20.


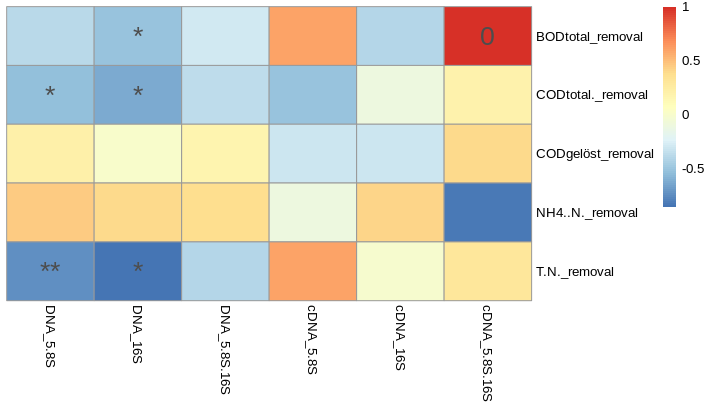


Supplementary Figure 6: Spearman correlation with p-values shown in the heatmap of 5.8S Rozellomycota and 16S qPCR results and water parameters, without multiple comparison correction.

Supplementary Figure 7: Spearman correlation between measured water parameters and eukaryotic abundance (18S DNA ASVs), without multiple comparison correction.


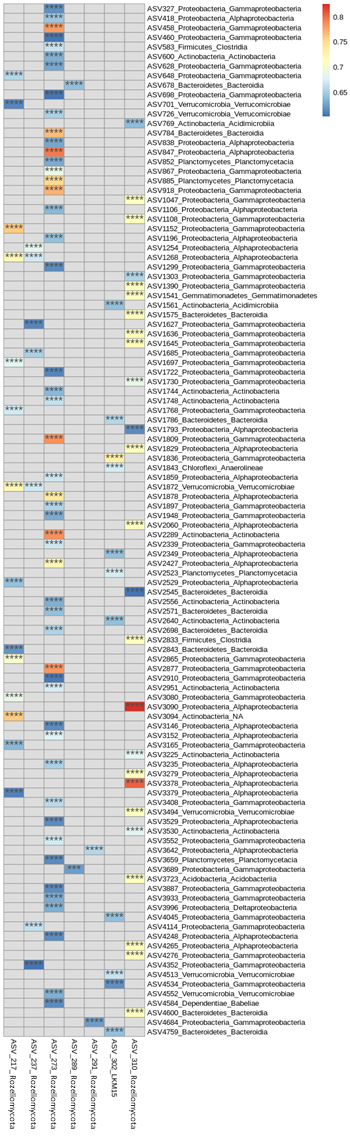

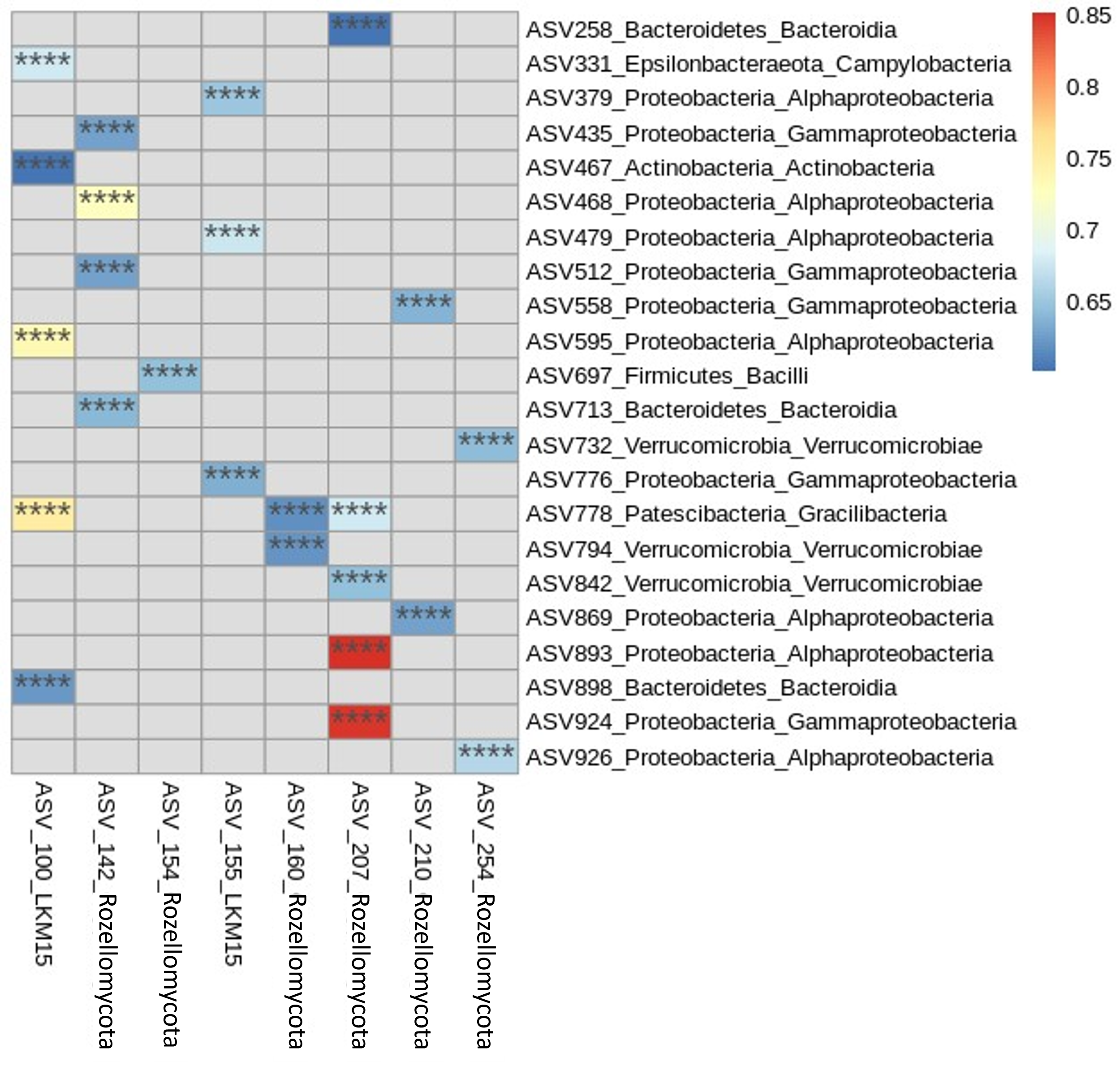


**A**

**B**

Supplementary Figure 8: Spearman correlation between Rozellomycota and bacteria cDNA and DNA (A, B, respectively) ASVs. A cutoff of r > 0.6 and a p-value < 0.05 was used, without multiple comparison correction.

Supplementary Table 5: Spearman correlation of 18S cDNA ASVs, a cutoff of r > 0.6 and a p-value < 0.05 was used, without multiple comparison correction.

|  | ASV7_Ciliophora | ASV36_Unclassified | ASV65_Nematoda |
| --- | --- | --- | --- |
| ASV5_Rozellomycota | 0.711 | 0.612 | 0 |
| ASV14_Rozellomycota | 0 | 0.622 | 0 |
| ASV38_Rozellomycota | 0 | 0.607 | 0 |
| ASV47_Rozellomycota | 0 | 0 | 0.70 |

Supplementary Table 6: Spearman correlation of 18S DNA ASVs, a cutoff of r > 0.6 and a p-value < 0.05 was used, without multiple comparison correction.

|  |  | *Ciliophora* | | | | | | | *Nematoda* | *Cercozoa* | *Ascomycota* | *Basidiomycota* | | *LKM*  *15* |
| --- | --- | --- | --- | --- | --- | --- | --- | --- | --- | --- | --- | --- | --- | --- |
| ASV | | 3 | 4 | 21 | 26 | 56 | 65 | 80 | 14 | 24 | 35 | 125 | 128 | 148 |
| *Rozellomycota* | 7 | - | - | - | - | - | - | - | - | - | 0.61 | - | - | - |
|  | 36 | 0.65 | - | - | - | - | - | - | - | - | - | - | - | 0.73 |
|  | 66 | - | -0.61 | - | -0.60 | - | 0.61 | - | 0.61 | 0.64 | 0.61 | - | - | - |
|  | 69 | - | - | -0.60 | - | - | - | - | - | - | - | - | - | - |
|  | 76 | - | - | - | - | - | - | 0.67 | - | - | - | - | 0.61 | - |
|  | 181 | - | - | - | - | 0.61 | - | - | - | - | - | 0.69 | - | - |
